# Measures of Longitudinal Immune Dysfunction and Risk of AIDS and Non-AIDS Defining Malignancies in Antiretroviral-Treated People With Human Immunodeficiency Virus

**DOI:** 10.1093/cid/ciad671

**Published:** 2023-12-13

**Authors:** Frédérique Chammartin, Amanda Mocroft, Alexander Egle, Robert Zangerle, Colette Smith, Cristina Mussini, Ferdinand Wit, Jörg Janne Vehreschild, Antonella d’Arminio Monforte, Antonella Castagna, Laurent Bailly, Johannes Bogner, Stéphane de Wit, Raimonda Matulionyte, Matthew Law, Veronica Svedhem, Joan Tallada, Harmony P Garges, Andrea Marongiu, Álvaro H Borges, Nadine Jaschinski, Bastian Neesgaard, Lene Ryom, Heiner C Bucher, for the RESPOND Study Group, F Wit, M van der Valk, M Hillebregt, K Petoumenos, M Law, R Zangerle, H Appoyer, C Stephan, M Bucht, N Chkhartishvili, O Chokoshvili, A d’Arminio Monforte, A Rodano, A Tavelli, I Fanti, J Casabona, J M Miro, J M Llibre, A Riera, J Reyes-Urueña, C Smith, F Lampe, A Sönnerborg, K Falconer, V Svedhem, H Günthard, B Ledergerber, H Bucher, K Kusejko, J C Wasmuth, J Rockstroh, J J Vehreschild, G Fätkenheuer, L Ryom, M Law, R Campo, S De Wit, H Garges, H Günthard, J Lundgren, I McNicholl, J Rooney, C Smith, V Vannappagari, G Wandeler, L Young, R Zangerle, J Lundgren, H Günthard, J Begovac, A Bruguera, H Bucher, A Castagna, R Campo, N Chkhartishvili, A D’Arminio Monforte, N Dedes, H Garges, J Kowalska, M Law, I McNicholl, C Mussini, C Necsoi, L Peters, K Petoumenos, C Pradier, D Raben, J Rockstroh, J Rooney, L Ryom, C Smith, A Sönnerborg, C Stephan, V Vannappagari, J J Vehreschild, A Volny Anne, G Wandeler, J C Wasmuth, E D Williams, F Wit, L Young, R Zangerle, L Ryom, A Mocroft, B Neesgaard, L Greenberg, N Jaschinski, A Timiryasova, L Bansi-Matharu, D Raben, L Peters, E Tusch, W Bannister, A Roen, D Byonanebye, O Fursa, A Pelchen-Matthews, J Reekie, V Svedhem-Johansson, M Van der Valk, F Wit, K Grabmeier-Pfistershammer, R Zangerle, J Hoy, M Bloch, D Braun, A Calmy, G Schüttfort, M Youle, S De Wit, C Mussini, S Zona, A Castagna, A Antinori, N Chkhartishvili, N Bolokadze, E Fontas, K Dollet, C Pradier, J M Miro, J M Llibre, J J Vehreschild, C Schwarze-Zander, J C Wasmuth, J Rockstroh, K Petoumenos, J Hutchinson, M Law, J Begovac, C Duvivier, G Dragovic, R Radoi, C Oprea, M Vasylyev, J Kowalska, R Matulionyte, V Mulabdic, G Marchetti, E Kuzovatova, N Coppola, I Aho, S Martini, H Bucher, A Harxhi, T Wæhre, A Pharris, A Vassilenko, G Fätkenheuer, J Bogner, A Maagaard, E Jablonowska, D Elbirt, G Marrone, C Leen, C Wyen, L Dahlerup Rasmussen, C Hatleberg, M Kundro, N Dedes, E Dixon Williams, J Gallant, C Cohen, M Dunbar, A Marongiu, V Vannappagari, H Garges, R Campo, L Young, A Volny Anne, N Dedes, L Mendao, E Dixon Williams, N Jaschinski, B Neesgaard, A Timiryasova, O Fursa, O Valdenmaier, J F Larsen, M Gardizi, D Raben, L Peters, L Ryom, T W Elsing, L Ramesh Kumar, S Shahi, K Andersen, J Reekie, L Greenberg, L Bansi-Matharu, K Petoumenos, D Byonanebye, E Tusch, A Roen, W Bannister, A Mocroft

**Affiliations:** Division of Clinical Epidemiology, Department of Clinical Research, University Hospital Basel and University of Basel, Basel, Switzerland; CHIP, Department of Infectious Diseases, Rigshospitalet, University of Copenhagen, Copenhagen, Denmark; Centre for Clinical Research, Epidemiology, Modelling and Evaluation (CREME), Institute for Global Health, University College London, London, United Kingdom; Austrian HIV Cohort Study (AHIVCOS), Paracelsus Medical University Hospital, Salzburg, Austria; Austrian HIV Cohort Study (AHIVCOS), Medizinische Universität Innsbruck, Innsbruck, Austria; The Royal Free HIV Cohort Study, Royal Free Hospital, University College London, London, United Kingdom; Modena HIV Cohort, Università degli Studi di Modena, Modena, Italy; AIDS Therapy Evaluation in the Netherlands (ATHENA) Cohort, HIV Monitoring Foundation, Amsterdam, The Netherlands; Department I of internal Medicine, University Hospital Cologne, Cologne, Germany; Italian Cohort Naive Antiretrovirals (ICONA), ASST Santi Paolo e Carlo, Milano, Italy; San Raffaele Scientific Institute, Università Vita-Salute San Raffaele, Milano, Italy; Nice HIV Cohort, Department of Public Health, Université Côte d’Azur—Centre Hospitalier Universitaire de Nice, UR2CA, Nice, France; Division of Infectious Diseases, Medizinische Klinik und Poliklinik IV, LMU University Hospital, LMU Munich, Munich, Germany; CHU Saint-Pierre, Centre de Recherche en Maladies Infectieuses a.s.b.l., Brussels, Belgium; Vilnius University, Faculty of Medicine, Department of Infectious Diseases and Dermatovenerology; Vilnius University Hospital Santaros Klinikos, Vilnius, Lithuania; The Australian HIV Observational Database (AHOD), Kirby Institute, University of New South Wales, New South Wales, Australia; Division of Infectious Diseases, Department of Medicine, Karolinska Institute and Karolinska University Hospital, Stockholm, Sweden; European AIDS Treatment Group (EATG), Brussels, Belgium; ViiV Healthcare, Durham, North Carolina, USA; Gilead Sciences, Foster City, California, USA; CHIP, Department of Infectious Diseases, Rigshospitalet, University of Copenhagen, Copenhagen, Denmark; Department of Infectious Diseases Immunology, Statens Serum Institut, Copenhagen, Denmark; CHIP, Department of Infectious Diseases, Rigshospitalet, University of Copenhagen, Copenhagen, Denmark; CHIP, Department of Infectious Diseases, Rigshospitalet, University of Copenhagen, Copenhagen, Denmark; CHIP, Department of Infectious Diseases, Rigshospitalet, University of Copenhagen, Copenhagen, Denmark; Department of Infectious Diseases 144, Hvidovre University Hospital, Copenhagen, Denmark; Division of Clinical Epidemiology, Department of Clinical Research, University Hospital Basel and University of Basel, Basel, Switzerland

**Keywords:** CD4:CD8 ratio, HIV infection, malignancy, observational study, antiretroviral therapy

## Abstract

**Background:**

Human immunodeficiency virus (HIV) infection leads to chronic immune activation/inflammation that can persist in virally suppressed persons on fully active antiretroviral therapy (ART) and increase risk of malignancies. The prognostic role of low CD4:CD8 ratio and elevated CD8 cell counts on the risk of cancer remains unclear.

**Methods:**

We investigated the association of CD4:CD8 ratio on the hazard of non-AIDS defining malignancy (NADM), AIDS-defining malignancy (ADM) and most frequent group of cancers in ART-treated people with HIV (PWH) with a CD4 and CD8 cell counts and viral load measurements at baseline. We developed Cox proportional hazard models with adjustment for known confounders of cancer risk and time-dependent cumulative and lagged exposures of CD4:CD8 ratio to account for time-evolving risk factors and avoid reverse causality.

**Results:**

CD4:CD8 ratios below 0.5, compared to above 1.0, were independently associated with a 12-month time-lagged higher risk of ADM and infection-related malignancies (adjusted hazard ratio 2.61 [95% confidence interval {CI }1.10–6.19] and 2.03 [95% CI 1.24–3.33], respectively). CD4 cell counts below 350 cells/μL were associated with an increased risk of NADMs and ADMs, as did infection, smoking, and body mass index-related malignancies.

**Conclusions:**

In ART-treated PWH low CD4:CD8 ratios were associated with ADM and infection-related cancers independently from CD4 and CD8 cell counts and may alert clinicians for cancer screening and prevention of NADM.

## Teaser

Low CD4:CD8 ratios are independently of CD4 cell counts associated with increased risk of AIDS-defining and infection-related malignancies in antiretroviral-treated people with human immunodeficiency virus (HIV). Combined with cancer screening strategies, markers' monitoring could benefit individuals wiht inadequate immunological reconstitution.

Modern antiretroviral therapy (ART) has led to a major reduction in AIDS events and a drastic increase in life expectancy of people with human immunodeficiency virus (HIV, PWH) [1]. At the same time, HIV care providers are facing a growing number of PWH with serious comorbidities. Among these, non-AIDS defining malignancies (NADM) represent the most frequent condition and are now a major underlying cause of death in PWH [2, 3]. The risk of NADM in PWH is multifactorial and is particularly impacted by highly prevalent oncogenic viral co-infections and smoking [4–6].

HIV-induced immune activation and inflammation has been suggested as another important driver for the growing incidence of NADM [7, 8]. Pathophysiological mechanisms involve the immune response to the virus itself, the production of pro-inflammatory cytokines, the expression of HIV gene products by activated lymphocytes and macrophages, the immunodeficiency-induced reactivation and replication of other viruses or the translocation of intestinal bacterial flora into the systemic circulation [9, 10]. Chronic immune activation can persist even among virally suppressed PWH, leading to an exhaustion of the immune resources, mimicking the process of aging-associated immune-senescence and installing an immune dysfunction.

The CD4:CD8 ratio represents a surrogate marker of defective T lymphocytes in HIV infection, which in recent years has regained attention [11, 12]. Expansion of CD8 cell counts with the consequence of a low ratio, in particular in well-treated and virologically suppressed individuals, may characterize a subpopulation with distinct immunological abnormalities and chronic inflammation [13]. Some authors have concluded that ART treated patients with high CD4 cell counts but low CD4:CD8 ratio may characterize a population at risk of immuno-senescence [14, 15].

Whether CD4:CD8 ratio or CD8 cell counts may represent markers for innate and adaptive immune activation that are predictive for specific type of cancers requires additional evidence. Addressing these issues in a large observational study is important since the identification of PWH at high risk of malignancies should lead to better patient management by reinforcing prevention programs and screening strategies. For example, low-dose computed tomography screening showed a reduction in lung-cancer mortality in high-risk population [16], and active monitoring together with lesions treatment reduces the risk of anal cancer among PWH with high grade squamous intraepithelial lesions [17]. Here we assess whether CD4:CD8 ratio and CD8 cell counts may be useful biomarkers for the risk of NADM, AIDS-defining malignancies (ADM) and specific cancers, after accounting for known risk factors of cancers and sociodemographic participant characteristics.

## METHODS

This prospective observational cohort study is based on data from the international cohort consortium of infectious diseases (RESPOND) [18], a collaboration of 17 observational studies across Europe and Australia including more than 30 000 PWH. CD4 and CD8 cell counts, human immunodeficiency (HI) viral load, information on ART, tobacco smoking, sociodemographic and behavioural parameters and AIDS defining illnesses are recorded at enrollment and during routine biannual or annual follow-up visits. Clinical events such as ADM and NADM, as well as information on ART, smoking and other AIDS defining illnesses, are reported annually to a central coordinating centre using standard case report forms following HIV Cohorts Data Exchange Protocol for data collection (https://hicdep.org/). Prospective follow-up started on 1 October 2017, with retrospective data collected back to 1 January 2012. Cancer events occurring during the RESPOND validation period are centrally adjudicated by clinicians at the RESPOND coordinating centre using pre-specified algorithms as detailed elsewhere [19]. Participants consented to share data with RESPOND according to local or national requirements and all cohorts had to approve the sharing of data with RESPOND. Ethical approval was obtained, if required, from the relevant bodies for collection and sharing of data. Data are stored on secure servers at the RESPOND coordinating centre in Copenhagen, Denmark, in accordance with current legislation and under approval by the Danish Data Protection Agency (approval number 2012-58-0004, RH-2018-15, 26/1/2018), under the EU General Data Protection Regulation (2016/579).

### Study Population

Our analysis includes ART treated PWH above 18 years followed between 1 January 2012 (date from which cancers were retrospectively collected) and 31 December 2020 (date of the database closure). We excluded PWH with no CD4 and CD8 cell counts or viral load measurements at baseline, a record of any malignancy prior to baseline or in the first 12 months of follow-up, and individuals with a follow-up time below 12 months. Baseline is defined as the later of cohort entry and 1 January 2012.

### Clinical Outcome

Our primary outcome is the occurrence of a first incident NADM. Secondary outcomes include ADM (non-Hodgkin lymphoma, Kaposi's sarcoma and invasive cervical cancer), and infection-related cancers (non-Hodgkin lymphoma, Kaposi sarcoma, Hodgkin lymphoma, and liver, invasive anal, cervical, penile, oesophageal, and stomach cancers), smoking-related malignancy (lung, head and neck, bladder, pancreas, colorectal, liver, kidney, rectum, cervical, oesophageal, lip, and stomach cancers) and body mass index (BMI)-related malignancy (pancreas, colorectal, liver, breast, kidney, rectum, gall bladder, oesophageal and thyroid cancers). Grouping of the different cancers was discussed with a panel of external oncologists at the initiation of the RESPOND consortium and defined by the RESPOND cancer working group [19, 20] for consistent use in all cohort projects explicitly allowing for not mutually exclusive classification.

### Baseline and Time-Updated Exposure Variables

We considered immunological (CD4 cells and CD8 cell counts, CD4:CD8 ratio) and HI viral load factors as time-updated exposure variables. In particular, time-updated exposures are considered as point exposures observed 12 months prior to a given follow-up (12 months lagged exposure) to avoid risks of detecting associations that reflect reverse causality. Baseline sociodemographic individual characteristics include sex (male/female), age, risk group (men having sex with men [MSM], people who inject drugs [PWID], and heterosexual/other/unknown), and race (White and other/unknown). Smoking (yes/no) is regarded as a time-varying lagged exposure at 12 months. Obesity (BMI >30 kg/m^2^), hepatitis B (positive surface antigen or detectable DNA) and C status (positive antibody test or detectable RNA) are also considered at baseline.

### Statistical Analysis

We aimed to assess the association of immunological and viral factors on the hazard of malignancies using Cox models with time-dependent covariates after adjusting for the baseline socio-demographic patient characteristics, sex, age, risk, and race. Infection-related malignancy analysis was further adjusted for hepatitis C and B status, whereas smoking-related and BMI related malignancy analyses were additionally adjusted for 12 months lagged smoking status and baseline obesity, respectively. We modeled time from 12 months after baseline to the first diagnosis of the considered malignancies in the analysis, death, or cohort administrative censoring, whichever came first. The first 12 months of follow-up were thus removed from the risk set to remove immortal bias induced by our study design. Time-updated variables were updated at the end of each follow-up month and missing information was extrapolated using the last observation carried forward (LOCF). Thus, at each follow-up month, participants who have experienced an event are compared with those currently at risk.

We assessed the robustness of our results in sensitivity analyses by considering time-updated exposure at different lags. In particular, we additionally considered point exposures lagged at 24 and 36 months, as well as cumulative exposures using simple moving averages (SMA) over the past 12 months and 24 months (12 months SMA are additionally lagged at 12 and 24 months, whereas 24 months SMA were lagged at 12 months) and baseline exposures. Additional sensitivity analyses included LOCF restricted to a maximum of 12 months. Hence, when gaps between laboratory measurements were longer than 12 months, the laboratory measurement for this specific month was considered as missing, and events observed during this specific follow-up month were excluded from the analysis. This implies that cancer diagnosis occurring during these months were censored. For smoking-related malignancy, we also performed a sensitivity analysis by excluding cohort for which smoking information was missing for more than 30% of the overall participants. All analyses were done in R Project for Statistical Computing (version 4.1.0) software [21]. Data management was performed in Stata SE 17-VA [22].

## RESULTS

**Figure 1.**
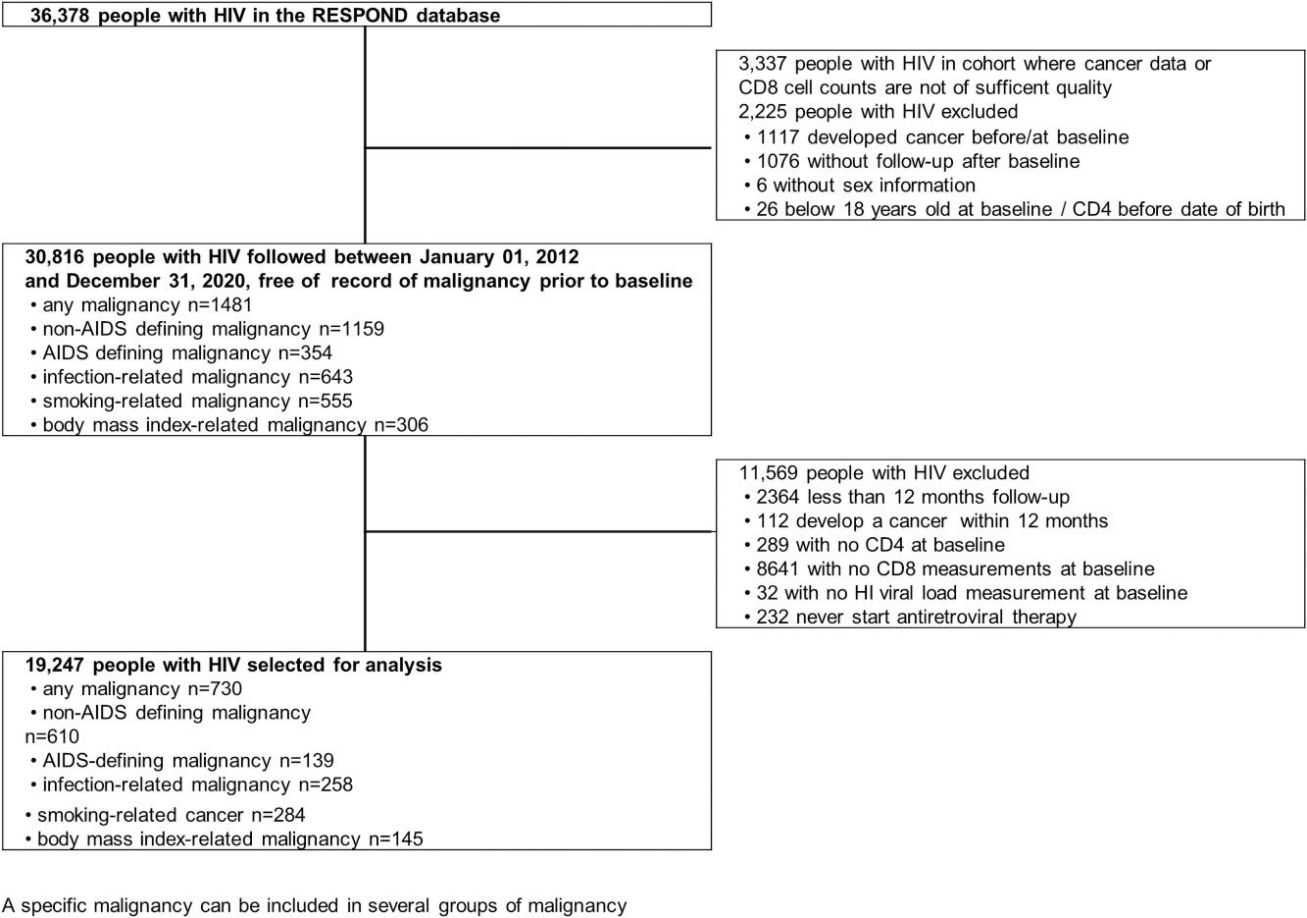
Flow chart of people with HIV selection and overview of number of specific type of malignancy. Abbreviation: HIV, human immunodeficiency virus.

A total of 30 816 PWH above 18 years of age were followed after 1 January 2012 onward without history of a malignancy at baseline. Malignancy, irrespective of the type, was diagnosed in 1481 individuals over 183 406 person-years (PY) (incidence rate [IR] 8.08 per 1000 PY (95% confidence interval [CI] 7.67–8.50). NADM and ADM had an IR of 6.32 per 1000 PY (95% CI 5.96–6.69) and 1.93 per 1000 PY (95% CI 1.74–2.14), respectively. In total, 11 569 individuals had no CD4 and CD8 cell counts or viral load measurements recorded after January 2012, never started ART, or had < 12 months follow-up and were excluded, leaving 19 247 individuals and 730 malignancies for analysis (Figure 1). Seventy-five percent of the dropped individuals were excluded due to missing CD8 laboratory measurements; the majority (80%) being from one large cohort that only measured CD8 cell counts at enrollment and after that only for HIV/hepatitis C coinfected individuals. Further details on the classification and frequency of single malignancies are provided in Table 1.

**Table 1.** Types and Frequency of First Cancers Diagnosed for Each Malignancy Group Among People With HIV With CD4 Cells, CD8 Cells and HI Viral Load at Baseline

Non-AIDS Defining			AIDS Defining			Smoking-Related		
Malignancy	n	%	Malignancy	n	%	Malignancy	n	%
Lung	73	11.97	Non-Hodgkin lymphoma	73	52.52	Lung	78	27.46
Anal	60	9.84	Kapsoi sarcoma	56	40.29	Head and neck cancer^a^	37	13.03
Prostate	54	8.85	Cervical	10	7.19	Bladder	34	11.97
Malignant melanoma	41	6.72	*Total*	*139*	*100*.*00*	Pancreas	32	11.27
Head and neck cancer	40	6.56	Infection-related	…	…	Colorectal	27	9.51
Bladder	32	5.25	malignancy	n	%	Liver	24	8.45
Pancreas	31	5.08	Non-Hodgkin lymphoma	71	19.19	Kidney	16	5.63
Colorectal	26	4.26	Anal	60	16.22	Rectum	12	4.23
Hodgkin lymphoma	24	3.93	Kaposi sarcoma	55	14.86	Cervical	10	3.52
Liver	24	3.93	Liver	24	6.49	Oesophageal	6	2.11
Breast	16	2.62	Hodgkin lymphoma	22	5.95	Lip	6	2.11
Kidney	16	2.62	Cervical	10	2.70	Stomach	2	0.70
Gynecological	15	2.46	Penile	8	2.16	*Total*	*284*	*100*.*00*
Rectum	12	1.97	Oesophagal	6	1.62	Body mass index-	…	…
Connective tissue	8	1.31	Stomach	2	0.54	related		
Penile	8	1.31	*Total*	*258*	*100*.*00*	malignancy	n	%
Testicular	8	1.31	…	…	…	Pancreas	32	22.07
Gall bladder	7	1.15	…	…	…	Colorectal	28	19.31
Oesophageal	6	0.98	…	…	…	Liver	24	16.55
Leukemia	6	0.98	…	…	…	Breast	17	11.72
Lip	5	0.82	…	…	…	Kidney	16	11.03
Multiple myeloma	4	0.66	…	…	…	Rectum	12	8.28
Brain	2	0.33	…	…	…	Gall bladder	7	4.83
Stomach	2	0.33	…	…	…	Oesophageal	6	4.14
Bone	1	0.16	…	…	…	Thyroid	3	2.07
Other/unclassified	89	14.59	…	…	…	*Total*	*145*	*100*.*00*
*Total*	*610*	*100*.*00*	…	…	…	…	…	…

Abbreviation: HIV, human immunodeficiency virus.

^a^Smoking-related head and neck malignancies include oral cavity, hypo- and oro-pharyngeal, laryngeal, saliva gland, sino/nasal cavity, and unspecified sub-types. Each cancer represents the first cancer diagnosed within the group.

Baseline sociodemographic and clinical characteristics were compared between the overall population in the RESPOND database and those included in the final analysis (Supplementary Table 1). They were found to be similar. Baseline characteristics of individuals included in the analysis and of individuals with specific incident cancers are provided in Table 2. PWH who developed cancers were generally older compared to PWH included in the analysis. Individuals of White race current smokers, with a positive baseline hepatitis C status, and PWID were more prevalent among individuals who developed a smoking-related malignancy. BMI-related malignancies were observed more frequently in heterosexuals, females, and obese individuals, whereas males and individuals with positive hepatitis B status were more prevalent among individuals that developed infection-related malignancy. The median time on ART at cancer diagnosis varies from 9.1 years (95% CI 4.3–16.4) for ADM to 15.0 years (95% CI 8.8–19.0) for smoking-related malignancies, suggesting that individuals included in our analysis that developed malignancies were mainly long-term treated PWH.

**Table 2.** Baseline Characteristics of Patients With CD4 Cells. CD8 Cells and HI Viral Load at Baseline, Overall and for Patients With Incident Cancers

	PWH Included in the Analysis	PWH With Non-AIDS Defining Malignancy	PWH With AIDS Defining Malignancy	PWH With Infection-Related Malignancy	PWH With Smoking-Related Malignancy	PWH With Body Mass Index-Related Malignancy
	(n = 19 247)	(n = 610)	(n = 139)	(n = 258)	(n = 284)	(n = 145)
Age [y]						
<50	13 698 (71.2%)	229 (37.5%)	84 (60.4%%)	139 (53.9%%)	98 (34.5%%)	61 (42.1%%)
50–65	4746 (24.7%)	302 (49.5%%)	46 (33.1%%)	100 (38.8%%)	151 (53.2%)	70 (48.3%)
≥65	803 (4.2%)	79 (13.0%)	9 (6.5%)	19 (7.4%)	35 (12.3%)	14 (9.7%)
Median age (interquartile range) [y]	44 (36–51)	52 (47–59)	46 (38–52)	49 (42–55)	52 (47–59)	51 (46–57)
Sex						
Male	14 715 (76.5%)	484 (79.3%)	108 (77.7%)	213 (82.6%)	223 (78.5%)	110 (75.9%)
Female	4532 (23.5%)	126 (20.7%)	31 (22.3%)	45 (17.4%)	61 (21.5%)	35 (24.1%)
Risk group						
Men having sex with men	9596 (49.9%)	267 (43.8%)	75 (54.0%)	134 (51.9%)	100 (35.2%)	51 (35.2%)
People who inject drugs	1921 (10.0%)	111 (18.2%)	14 (10.1%)	31 (12.0%)	76 (26.8%)	33 (22.8%)
Heterosexual	6544 (34.0%)	195 (32.0%)	39 (28.1%)	74 (28.7%)	91 (32.0%)	52 (35.9%)
Other/unknown	1186 (6.2%)	37 (6.1%)	11 (7.9%)	19 (7.4%)	17 (6.0%)	9 (6.2%)
Race						
White	12 934 (67.2%)	477 (78.2%)	92 (66.2%)	181 (70.2%)	233 (82.0%)	107 (73.8%)
Other/unknown	6313 (32.8%)	133 (21.8%)	47 (33.8%)	77 (29.8%)	51 (18.0%)	38 (26.2%)
CD4 cell count [cells/μL]						
<350	4731 (24.6%)	165 (27.0%)	56 (40.3%)	95 (36.8%)	78 (27.5%)	36 (24.8%)
350–500	4233 (22.0%)	127 (20.8%)	34 (24.5%)	59 (22.9%)	58 (20.4%)	33 (22.8%)
≥500	10 283 (53.4%)	318 (52.1%)	49 (35.3%)	104 (40.3%)	148 (52.1%)	76 (52.4%)
CD8 cell count [cells/μL]						
<1000	12 533 (65.1%)	386 (63.3%)	77 (55.4%)	148 (57.4%)	181 (63.7%)	91 (62.8%)
≥1000	6714 (34.9%)	224 (36.7%)	62 (44.6%)	110 (42.6%)	103 (36.3%)	54 (37.2%)
CD4:CD8 ratio						
<0.5	7271 (37.8%)	238 (39.0%)	80 (57.6%)	140 (54.3%)	114 (40.1%)	57 (39.3%)
0.5–1.0	8119 (42.2%)	247 (40.5%)	42 (30.2%)	83 (32.2%)	115 (40.5%)	57 (39.3%)
≥1	3857 (20.0%)	125 (20.5%)	17 (12.2%)	35 (13.6%)	55 (19.4%)	31 (21.4%)
HI viral load [copies/mL]						
<200*	12 590 (65.4%)	485 (79.5%)	75 (54.0%)	167 (64.7%)	233 (82.0%)	119 (82.1%)
≥200	6657 (34.6%)	125 (20.5%)	64 (46.0%)	91 (35.3%)	51 (18.0%)	26 (17.9%)
Body mass index [kg/m^2^]						
<30	12 785 (66.4%)	435 (71.3%)	81 (58.3%)	168 (65.1%)	214 (75.4%)	110 (75.9%)
≥30	1128 (5.9%)	40 (6.6%)	6 (4.3%)	17 (6.6%)	19 (6.7%)	14 (9.7%)
Missing	5334 (27.7%)	135 (22.1%)	52 (37.4%)	73 (28.3%)	51 (18.0%)	21 (14.5%)
Hepatitis C status						
Negative	14 029 (72.9%)	406 (66.6%)	94 (67.6%)	169 (65.5%)	170 (59.9%)	93 (64.1%)
Positive	2981 (15.5%)	152 (24.9%)	19 (13.7%)	50 (19.4%)	95 (33.5%)	45 (31.0%)
Unknown	2237 (11.6%)	52 (8.5%)	26 (18.7%)	39 (15.1%)	19 (6.7%)	7 (4.8%)
Hepatitis B status						
Negative	16 029 (83.3%)	532 (87.2%)	106 (76.3%)	203 (78.7%)	255 (89.8%)	131 (90.3%)
Positive	735 (3.8%)	29 (4.8%)	7 (5.0%)	18 (7.0%)	15 (5.3%)	8 (5.5%)
Unknown	2483 (12.9%)	49 (8.0%)	26 (18.7%)	37 (14.3%)	14 (4.9%)	6 (4.1%)
Smoking						
No	7043 (36.6%)	236 (38.7%)	50 (36.0%)	95 (36.8%)	89 (31.3%)	67 (46.2%)
Yes	6018 (31.3%)	225 (36.9%)	32 (23.0%)	73 (28.3%)	128 (45.1%)	48 (33.1%)
Unknown	6186 (32.1%)	149 (24.4%)	57 (41.0%)	90 (34.9%)	67 (23.6%)	30 (20.7%)
Median time on ART at diagnosis						
(interquartile range) [y]	…	13.7 (7.8–19.3)	9.1 (4.3–16.4)	12.3 (5.8–18.4)	15.0 (8.8–19.0)	14.9 (9.1–19.0)

Positive hepatitis C is defined as detectable RNA or a positive immunoglobulin G (IgG) antibody test; positive hepatitis B is defined as surface antigen positivity.

Abbreviations: ART, antiretroviral therapy; PWH, people with human immunodeficiency virus (HIV).

**Figure 2.**
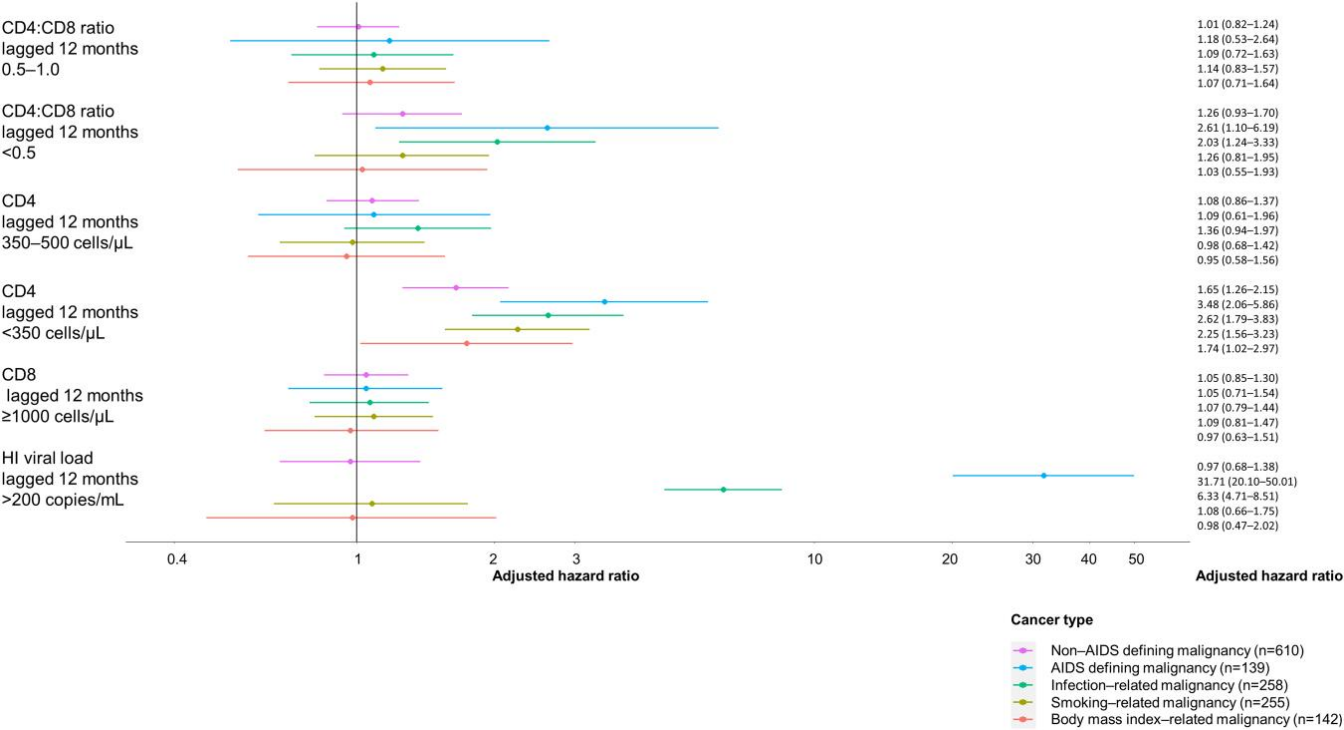
Adjusted hazard ratio of immunological and virological factors for non-AIDS defining, AIDS defining, infection, smoking and body mass related malignancies. All models are adjusted for age, sex, risk, and race. The model for infection-related malignancy is further adjusted for baseline chronic hepatitis C and B status. The model for smoking-related malignancy is further adjusted for 12 m lagged smoking status. The body mass index-related malignancy model is adjusted for baseline body mass index. Reference categories are CD4:CD8 ratio lagged 12 m ≥1.0, CD4 lagged 12 m ≥50 cells/μL, HI viral lead lagged 12 m ≤200 copies/mL. Abbreviation: HI, human immunodeficiency.

Figure 2 summarizes the adjusted hazard ratio (aHR) for cancer with 95% CI of immune function parameters that are lagged at 12 months (parameters' estimates of the full model are given in the Supplementary Table 2). The estimated mean aHR ranged from 1.03 (BMI-related malignancies) to 2.61 (AIDS-defining malignancies) when comparing CD4:CD8 ratios <0.5 to CD4:CD8 ratios >1.0. However, CD4:CD8 ratios <0.5 were significantly associated with infection-related malignancies (aHR 2.03; 95% CI 1.24–3.33) and AIDS-defining malignancies (aHR 2.61; 95% CI 1.10–6.19) when compared to CD4:CD8 ratios >1.0. CD4 cell counts <350 cells/μl increased the risk of NADMs (aHR: 1.65; 95% CI: 1.26–2.15), ADMs (aHR 3.48; 95% CI 2.06–5.86), infection-related malignancies (aHR 2.62; 95% CI 1.79–3.83), smoking-related malignancies (aHR 2.25; 95% CI 1.56–3.23) and BMI-related malignancies (aHR 1.74; 95% CI 1.02–2.97). In the adjusted analyses, none of the associations between CD8 cell counts and any of the malignancies analysed were statistically significant. HI viral loads ≥200 copies/mL were important drivers of ADM (aHR 31.71; 95% CI 20.10–50.01), and infection-related malignancy (aHR 6.33; 95% CI 4.71–8.51).

Sensitivity analyses results were robust for point lagged exposures at 24 and 36 months, as well as for several cumulative lagged exposures and baseline exposure (Supplementary Figure 1). When restricting the extrapolation of immunological and virological factors to 12 months, estimates from models were similar to models fitted on data with immunological and virological exposures that were extrapolated without the restriction of time. We therefore do not expect bias due to the extrapolation procedure (Supplementary Figure 2 vs Supplementary Figure 1). In addition, results for smoking-related malignancy were robust when excluding individuals from cohorts reporting suboptimal smoking information (data completeness lower than 70%) (Supplementary Figure 3), as well as to the exclusion of individuals from cohorts that did not routinely collect CD8 cell counts (Supplementary Figure 4), limiting the risk of selection bias. Finally, we considered smoking as a binary time-varying exposure rather than categorizing participants into current and past smokers. This did not affect our results, but we estimated in an additional sensitivity analysis not presented here a stronger association with current smoking status than previous smoking status for smoking-related malignancies, in line with past RESPOND analyses [19].

All final models satisfied the proportional assumption of the Cox regression model according to the test of temporal independence of Schoenfeld residuals. Multi-collinearity was not detected according to the variance inflation factors that were all below the threshold value of 5.

## DISCUSSION

In this large prospective multinational cohort study of ART treated individuals who were followed between 2012 and 2020, a low CD4:CD8 ratio was associated with significantly higher risk of ADM and infection-related cancers independent from CD4 cell counts. Elevated CD8 cell counts were not an important marker of any malignancy, whereas CD4 cell counts below 350 cells/μL were associated with a higher risk of all malignancies studied in this analysis. Unsuppressed HI viral load was associated with a substantial increase in risk of ADM and infection-related cancer, which also included ADM. Hence, altered immune function, as proxied by low CD4:CD8 ratio, might additionally affect the carcinogenesis of ADM and infection-related cancers and be an important additive marker to a low CD4 cell count for these malignancies among PWH.

Several cohort studies have investigated the association between CD4:CD8 ratio and the risk of non-AIDS defining malignancies and other clinical events [23–30]. Results are conflicting due to the lack of standardized methodology, different modelling approaches, the use of a mixture of combined endpoints that encompassed different disease entities, limitation of sample sizes, and missing smoking status data. For example, in the large ART-CC cohort, no association between CD4:CD8 ratio and non-AIDS related mortality was found, but this cohort lacked data on smoking status [23]. In the Italian ICONA cohort [24] and a Thai cohort [25] low CD4:CD8 ratio was associated with an increased risk of non-AIDS events, which included a composite endpoint of malignancies, cardiovascular, renal [25], and hepatic events [24]. However, the limited sample size did not allow differentiation among the risks for malignancy and for the remaining non-cancer events. The French APROCO cohort study was the first study to report a significant association between low CD4:CD8 ratio and increased risk of NADM [26]. A low CD4:CD8 cell ratio was associated with an increased risk of lung cancer [28] in the US Veteran cohort study, and a higher risk of Kaposi sarcoma and non-Hodgkin lymphoma in the COHERE cohort study [29]. In the Swiss HIV Cohort Study a low CD4:CD8 ratio was not associated with increased risk of anal, lung, prostate and liver cancers [27], whereas the North American AIDS Cohort Collaboration on Research and Design (NA-ACCORD) showed an association of low CD4 and CD8 ratio and the risk of lung, anal, and colorectal cancers in addition to the higher risk for the ADM non-Hodgkin lymphoma and Kaposi sarcoma [30]. Our study is important because it provides further evidence about an association between surrogate parameters of chronic immune activation in the presence of ART and the risk of NADMs, and in particular for infection-related cancers.

Strengths of this analysis are the large sample size with a large number of events grouping different centrally adjudicated malignancies and our ability to control for important known risk factors for cancers. In addition we considered virological and immunological factors under several functional forms to ensure the robustness of our results and present them in form that is directly useful for targeted patient management. Latency period in cancer studies can be subject to discussion. Here we primarily considered 12 month lagged exposure to exclude reverse causality while keeping sufficient statistical power for our analyses. The robustness of our results with regards to increased latency period suggest that immunological and virological factors appear to affect cancer risk early on and reflects long duration of cancers pathogenesis. In our modeling approach we used time-updated covariates, which enabled us to account for immunological and viral parameters that change over time. Therefore, missing laboratory measurements between periods had to be imputed, which could have made our approach prone to misclassification bias; however, a sensitivity analysis that restricted the extrapolation suggests it had little impact on our results.

Limitations of this study are that we did not additionally consider cubic splines that can capture more complex non-linear relationships [23] or weighted cumulative exposures that allow a flexible modelling time-dependent exposures [31]. Because our analysis looked at four different time-varying immunological and virological factors, a more systematic look at the complex relationship between all 4 parameters was not feasible also due to the limited number of events. In addition, we lacked information on bacterial pneumonia, cytomegalovirus (CMV), and human papillomavirus (HPV) infections. Also, alcohol consumption, which was not systematically collected by the majority of the cohorts involved in RESPOND could not be considered. Accounting for those factors would have reduced the risk of confounding bias in models that include associated cancers. Despite our efforts to adjust our models to the available risk factors, we cannot rule out bias due to unmeasured confounding. We note, however, that the effect of CD4:CD8 ratio on the risk of lung cancers remained after adjusting for bacterial pneumonia episodes in the US Veteran study [28]. Finally, CD4 and CD8 cell counts were the two available markers that routinely monitor the immune system. Data on additional markers of inflammation like plasma interleukin (IL)-6 or D-Dimer levels were not available and might add value in future studies.

## CONCLUSION

In this large cohort study with pooled data on PWH across Europe and Australia, a CD4 cell count of <350 cells/µL was associated with an increased risk of any studied malignancy. The study provides additional evidence that a low CD4:CD8 ratio carries an additional risk for ADM and infection-related malignancies. Therefore, regular monitoring of CD4 cells and CD4:CD8 ratios may provide benefit if it leads to enhanced cancer screening strategies for individuals who initiate ART late and do not achieve immune restoration above 350 cells/µL and >1.0, respectively. Our findings illustrate the importance of early HIV diagnosis and ART initiation with lifelong HIV suppression to reduce, in addition to other relevant clinical events, the risk of ADM and NADM. Whether PWH with insufficiently restored immune function profit on top of smoking cessation counselling from enhanced cancer screening programs should be further investigated.

## Supplementary Data

Supplementary materials are available at *Clinical Infectious Diseases* online. Consisting of data provided by the authors to benefit the reader, the posted materials are not copyedited and are the sole responsibility of the authors, so questions or comments should be addressed to the corresponding author.

## Supplementary Material

ciad671_Supplementary_Data

## Notes

***Author Contributions.*** F. C. and H. C. B. conceived and designed the study. F. C. did the statistical analysis. F. C. and H. C. B. verified the underlying data. H. C. B, and F. C. acquired funding for the statistical analysis. F. C. and H. C. B. drafted the manuscript, and all authors critically revised the manuscript and consented to final publication. All authors have full access to the data included in the study and accept responsibility for the decision to submit for publication.
