# Supplementary material for: Measures of Longitudinal Immune Dysfunction and Risk of AIDS and Non-AIDS Defining Malignancies in Antiretroviral-Treated People With Human Immunodeficiency Virus
Source: Clin Infect Dis. 2023 Dec 13;78(4):995–1004. doi: 10.1093/cid/ciad671 (PMC11006099; doi:10.1093/cid/ciad671)

Supplementary file to

Chammartin F et al. Measures of longitudinal immune dysfunction and risk of AIDS and non-AIDS defining malignancies in antiretroviral treated people with HIV

Table S1. Baseline socio-demographic and clinical characteristics of people with HIV (PWH) in the RESPOND database and PWH included in the analysis

	Overall RESPOND database (n=30816)	PWH included in the analysis (n=19247)
**Age [year]**		
< 50	21491 (69.7%)	13698 (71.2%)
50-65	7920 (25.7%)	4746 (24.7%)
≥ 65	1405 (4.6%)	803 (4.2%)
**Mean age (interquartile range)**	44 (36-51)	44 (36-51)
**Sex**		
Male	23350 (75.8%)	14715 (76.5%)
Female	7466 (24.2%)	4532 (23.5%)
**Risk group**		
Men having sex with men	14313 (46.4%)	9596 (49.9%)
People who inject drugs	4337 (14.1%)	1921 (10.0%)
Heterosexual	10128 (32.9%)	6544 (34.0%)
Other / unknown	2038 (6.6%)	1186 (6.2%)
**Race**		
White	22106 (71.7%)	12934 (67.2%)
Other / unknown	8710 (28.3%)	6313 (32.8%)
**CD4 cell count [cells/µl]**		
<350	5854 (23.5%)	4731 (24.6%)
350-500	5471 (22.0%)	4233 (22.0%)
≥ 500	13594 (54.5%)	10283 (53.4%)
Missing	5897	
**CD8 cell count [cells/µl]**		
<1000	12545 (65.1%)	12533 (65.1%)
≥ 1000	6721 (34.9%)	6714 (34.9%)
Missing	11550	
**CD4:CD8 ratio**		
<0.5	7277 (37.8%)	7271 (37.8%)
0.5-1.0	8126 (42.2%)	8119 (42.2%)
≥ 1	3861 (20.0%)	3857 (20.0%)
Missing	11552	
**HI viral load [copies/ml]**		
<200/detection limit	17320 (69.5%)	12590 (65.4%)
≥200	7609 (30.5%)	6657 (34.6%)
Missing	5887	
**Body mass index [kg/m^2]**		
<30	17177 (55.7%)	12785 (66.4%)
≥30	1471 (4.8%)	1128 (5.9%)
Missing	12168 (39.5%)	5334 (27.7%)
**Hepatitis C status**		
Negative	17270 (56.0%)	14029 (72.9%)
Positive	4959 (16.1%)	2981 (15.5%)
Unknown	8587 (27.9%)	2237 (11.6%)

**Hepatitis B status**		
Negative	20807 (67.5%)	16029 (83.3%)
Positive	1101 (3.6%)	735 (3.8%)
Unknown	8908 (28.9%)	2483 (12.9%)
**Smoking**		
No	9739(31.6%)	7043 (36.6%)
Yes	8290 (26.9%)	6018 (31.3%)
Unknown	12787(41.5%)	6186 (32.1%)

PWH: people with HIV

Positive hepatitis B = positive surface antigen or detectable DNA; positive hepatitis C = positive antibody test or detectable RNA

Table S2: Model parameter estimates for non-AIDS and AIDS defining malignancies and composite endpoints of infection-, smoking-, and body mass index-related malignancies

	**Non-AIDS defining malignancy**	**AIDS-defining malignancy**	**Infection-related malignancy**	**Smoking-related malignancy**	**Body mass index-related malignancy**
**CD4:CD8 ratio lagged 12 months**					
≥1.0	1	1	1	1	1
0.5-1.0	1.01 (0.82-1.24)	1.18 (0.53-2.64)	1.09 (0.72-1.63)	1.14 (0.83-1.57)	1.07 (0.71-1.64)
<0.5	1.26 (0.93-1.70)	2.61 (1.10-6.19)	2.03 (1.24-3.33)	1.26 (0.81-1.95)	1.03 (0.55-1.93)
**CD4 cell counts lagged 12 months (cells/µl)**					
≥500	1	1	1	1	1
350-500	1.08 (0.86-1.37)	1.09 (0.61-1.96)	1.36 (0.94-1.97)	0.98 (0.68-1.42)	0.95 (0.58-1.56)
<350	1.65 (1.26-2.15)	3.48 (2.06-5.86)	2.62 (1.79-3.83)	2.25 (1.56-3.23)	1.74 (1.02-2.97)
**CD8 cell counts lagged 12 months (cells/µl)**					
<1000	1	1	1	1	1
≥1000	1.05 (0.85-1.30)	1.05 (0.71-1.54)	1.07 (0.79-1.44)	1.09 (0.81-1.47)	0.97 (0.63-1.51)
**HI viral load lagged 12 months (copies(ml)**					
≤200/lower detection limit	1	1	1	1	1
>200	0.97 (0.68-1.38)	31.71 (20.10-50.01)	6.33 (4.71-8.51)	1.08 (0.66-1.75)	0.98 (0.47-2.02)
**Age (year)**	1.07 (1.07-1.08)	1.02 (1.01-1.04)	1.04 (1.03-1.05)	1.08 (1.07-1.09)	1.07 (1.05-1.08)
Sex					
Male	1	1	1	1	1
Female	0.97 (0.78-1.22)	1.31 (0.83-2.06)	0.89 (0.62-1.27)	0.91 (0.67-1.23)	0.89 (0.58-1.37)
**Risk group**					
Men having sex with men	1	1	1	1	1
People who inject drugs	1.98 (1.57-2.51)	0.62 (0.36-1.10)	0.67 (0.40-1.12)	4.56 (3.31-6.28)	3.95 (2.53-6.17)
Heterosexual	0.93 (0.75-1.14)	0.55 (0.34-0.90)	0.73 (0.53-1.02)	1.20 (0.86-1.67)	1.23 (0.79-1.93)
Other/unknown	1.23 (0.88-1.71)	1.03 (0.55-1.93)	1.14 (0.70-1.83)	1.52 (0.90-2.57)	1.42 (0.69-2.91)
**Race**					
White	1	1	1	1	1
Other/Unknown	0.83 (0.68-1.01)	1.07 (0.73-1.55)	1.14 (0.70-1.83)	0.79 (0.58-1.09)	1.07 (0.72-1.59)
**Hepatitis C status**					
Negative			1		
Positive			1.47 (0.97-2.25)		
Unknown			1.41 (0.88-2.26)		
**Hepatitis B status**					
Negative			1		
Positive			2.10 (1.34-3.29)		
Unknown			1.00 (0.62-1.62)		
**Smoking lagged 12 months**					
No				1	
Yes				1.77 (1.33-2.37)	
Unknown				1.11 (0.78-1.57)	
**Obesity (BMI>30 kg/m^2)**					
No					1
Yes					1.31 (0.75-2.29)
Unknown					0.59 (0.37-0.93)

Positive hepatitis B = positive surface antigen or detectable DNA; positive hepatitis C = positive antibody test or detectable RNA

Figure S1: Adjusted hazard ratio of immunological and virological factors for non AIDS defining, AIDS defining, infection-, smoking- and body mass-related malignancies in a sensitivity analysis that considers additional forms of exposures

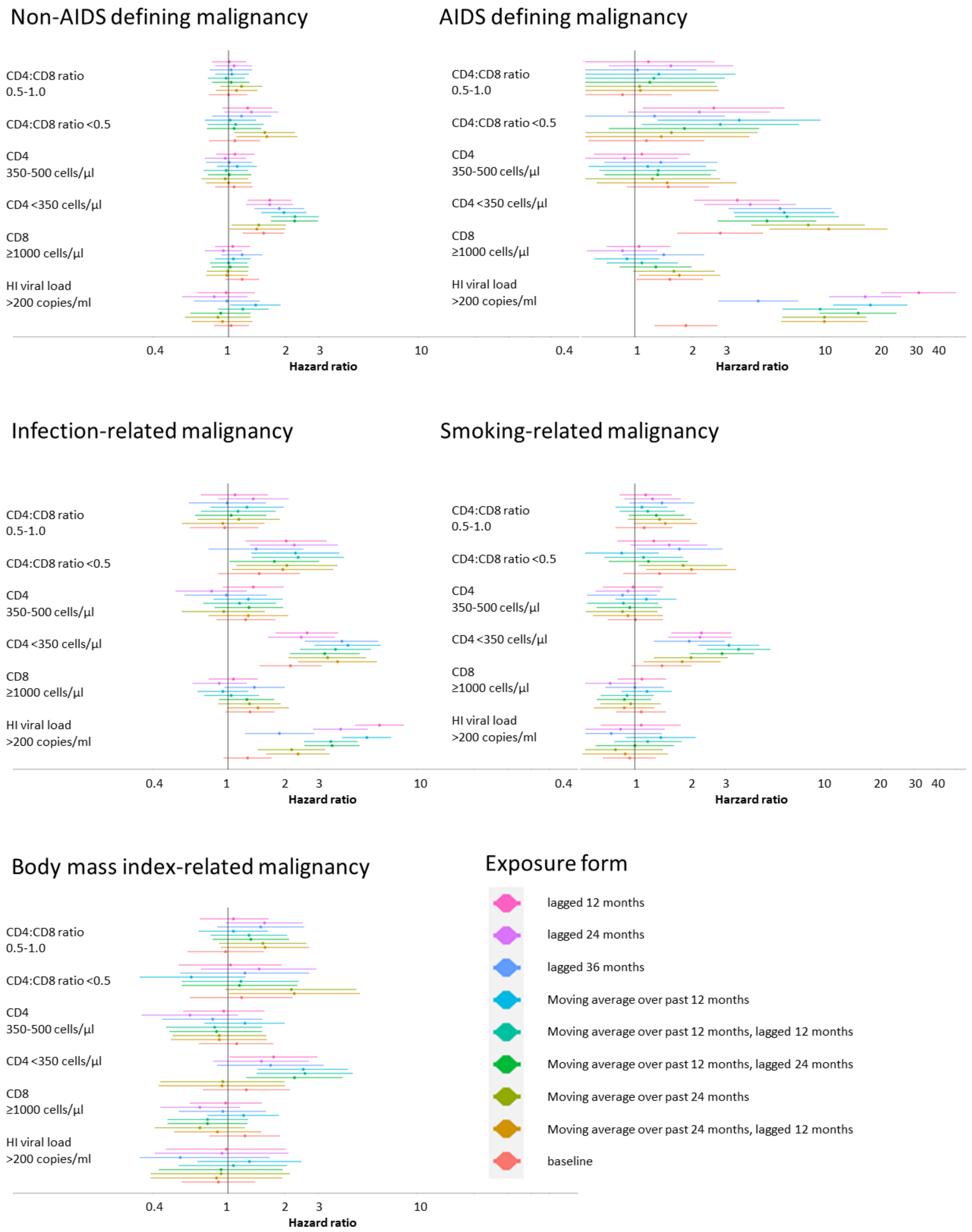

Figure S2: Adjusted hazard ratio of immunological and virological factors for non AIDS defining, AIDS defining, infection-, smoking-, and body mass-related malignancies in a sensitivity analysis that considers additional forms of exposures and restricted extrapolation of immunological and virological factors to 12 months

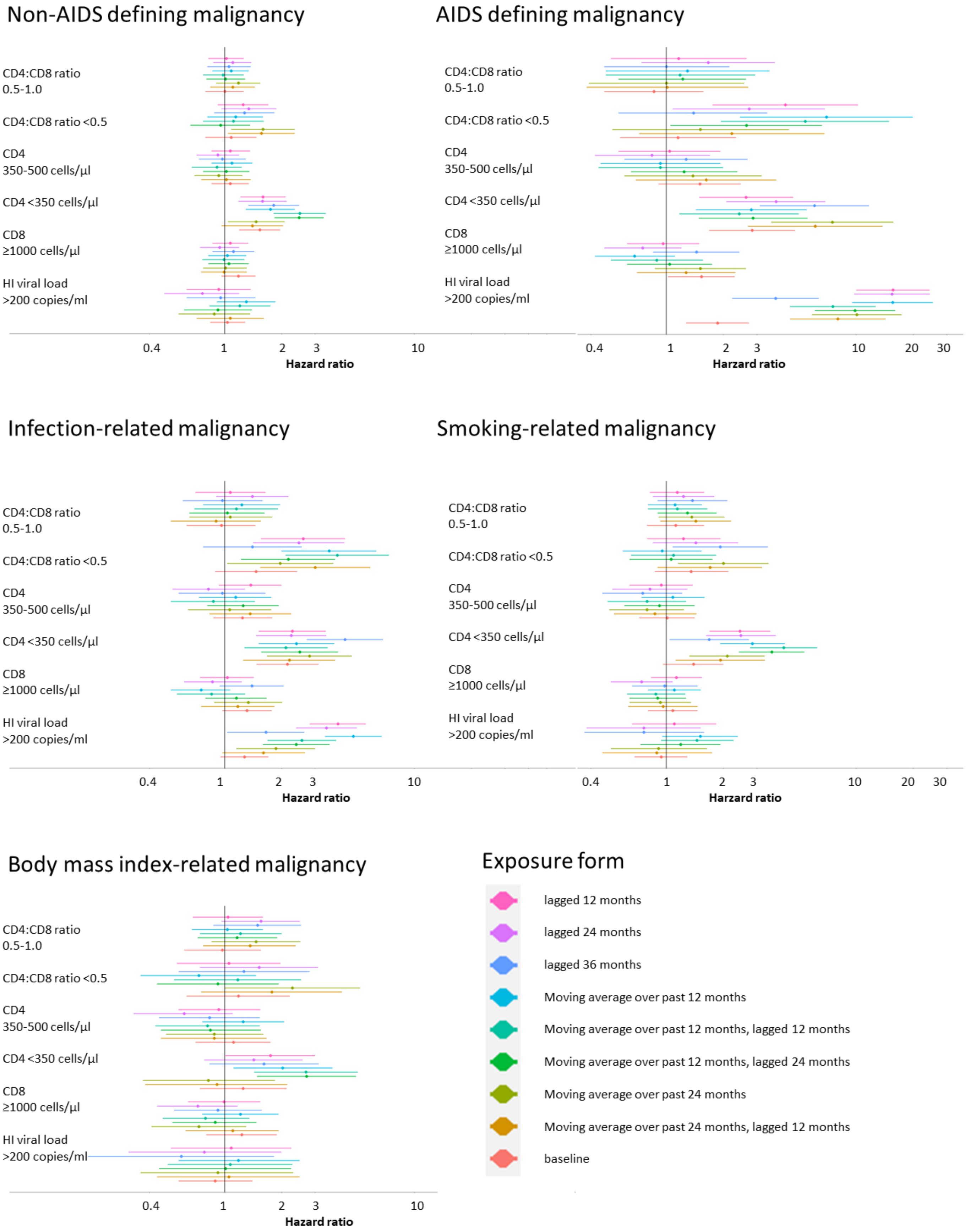

Figure S3: Adjusted hazard ratio of immunological and virological factors for smoking-related malignancies in a sensitivity analysis that excludes individuals from cohorts with insufficient smoking information

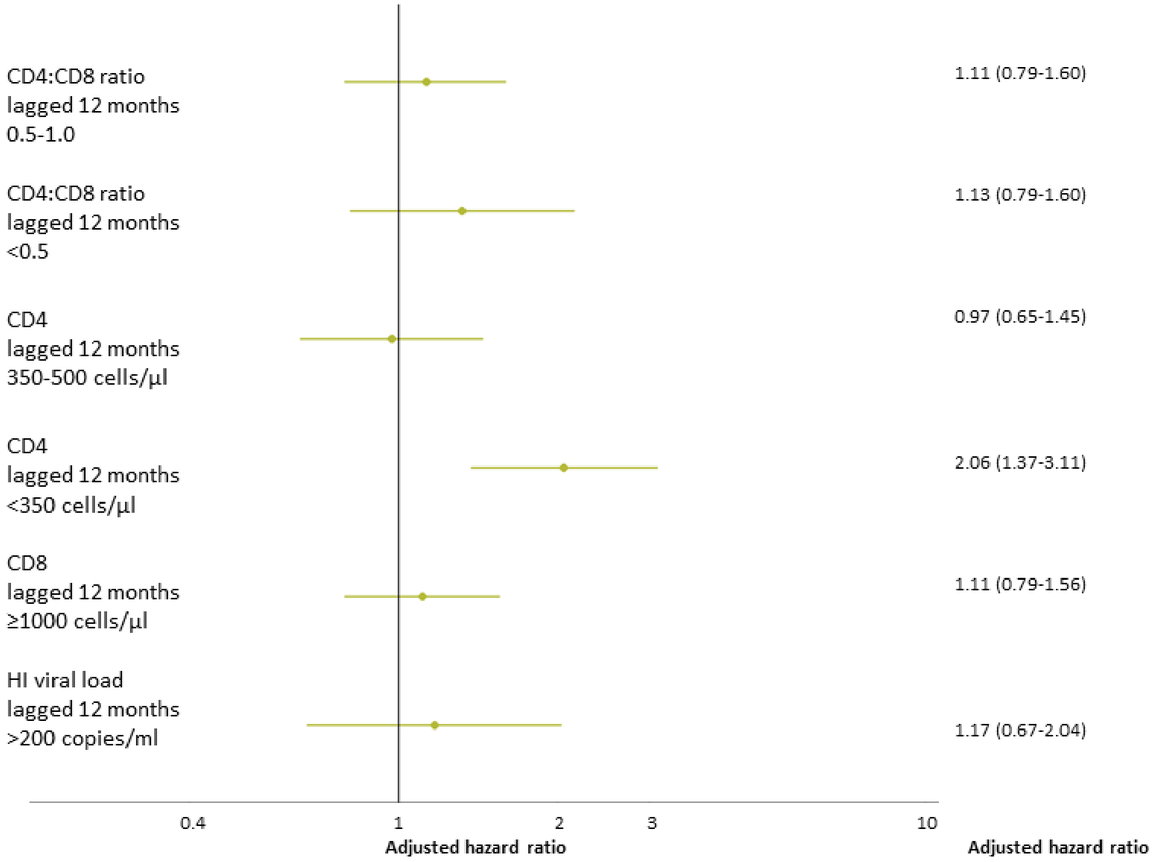

Figure S4: Adjusted hazard ratio of immunological and virological factors for smoking-related malignancies in a sensitivity analysis that excludes individuals from the EuroSIDA cohort that did not routinely collect CD8 cell counts

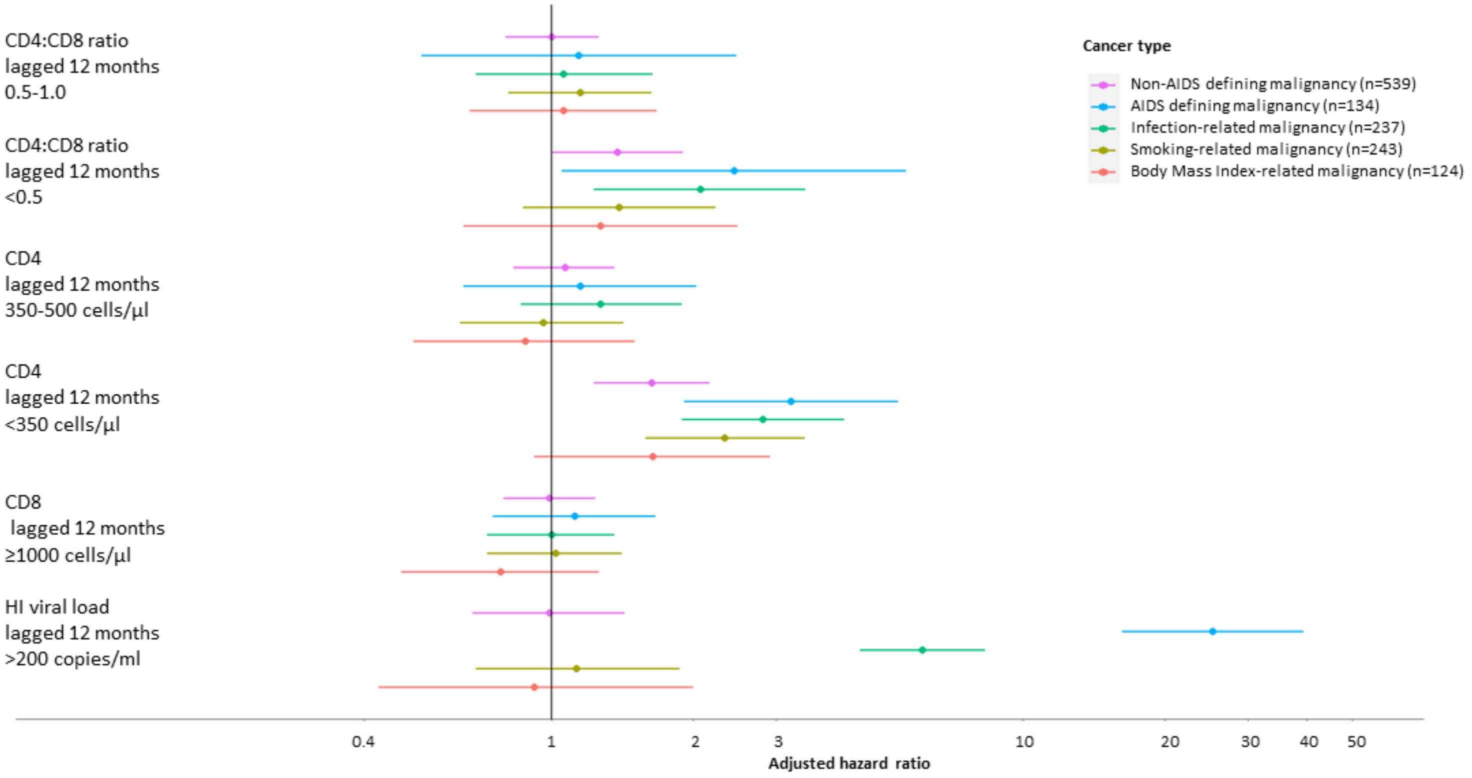

Supplement: ciad671_Supplementary_Data [file ciad671_supplementary_data.pdf]
